# Doctors Disagree

**Published:** 1887-01

**Authors:** 


### DOCTORS DISAGREE.

### THE TWO VIEWS.

A correspondent who signs himself Wilfred de Fonvielle, in an October number of the Parisian *Journal D' Hygiene*, makes a very spirited attack upon Henry Slade, the well-known American medium, whose haps and mishaps have given rise to much controversy on both sides of the Atlantic, during the last decade, and we deem it only fair that Hall's Journal of Health should open its columns to the other side of the question.

M. de Fonvielle's article is entitled "Horace et M. Slade," and reads as follows:

### HORACE AND MR. SLADE.

Translated from the *Journal d' Hygiene* (Paris).

The arrival of Mr. Slade in Paris has given an aftermath of actuality to a work which I published three or four years ago at the Drefous Library, under the title of "The Mountebanks of Science." In this little volume I gave an account of the manner of operating by the turn of the slate, whereby the operator acquired the reputation of a Fakir at the expense of my credulous countrymen.

The turning of the slate is very simple. The prestidigitator anticipates the questions which people usually ask and writes his answers beforehand upon the surface of the slate which he conceals underneath the table. He then brusquely presents his slate to his visitors, who expect a response from the spirits, and ignorant that the response has already been written, imagine that they are present and make use of the pencil left for them upon the prepared slate, which is found secured in the shallow space formed by its projecting frame and the face of the table.

The art of the prestidigitator consists in diverting the attention of those in waiting at the moment when he writes the response. To this end, he engages them in conversation; amuses them with various comic picture books; makes strange noises in his throat; taps them upon the shins with his foot; touches their hands; in a word, resorts to infinite trifles employed by the sharper in his specialities. They are indeed of a piece with those practised upon theatrical boards by professional jugglers.

Mr. Slade has been caught in *flagrante delicto* by Dr. Lankaster, who seized the slate in his hands at the moment when the operator invoked the spirits. This he followed up by a prosecution before a tribunal of justice, in which judgment was had against Slade.

Hastily quitting London when too hot for him, where he had already burnt his fingers, he sought refuge in Berlin. There he fell in with an ambitious (gallophobe) astronomer named Dr. Zollner, who had a "crow to pick" with Sir William Thompson, Tyndall and other celebrated English scientists, apropos of the theories of Faraday and Maxwell. To sustain his position, it became important for Dr. Zollner to prove the existence of a *fifth dimension.* He imagined that the experiences of Mr. Slade, proved the existence in space of a fifth dimension, and in this behalf wrote four large volumes in 8vo. of 500 to 600 pages each.

His experiments with Mr. Slade are described with great minuteness of detail, accompanied with photographic illustrations of slates containing a number of *fac similes* of spirit writing.

In Berlin, Slade practised the art of untying knots in cords, the two ends whereof were spliced together and sealed with wax stamped with the seal of Dr. Zollner, thus ensuring good faith.

These extraordinary experiments made so much noise that they were subsequently repeated in St. Petersburg under the eyes of the Grand Duke Constantine.

I resign all this to the *quacks in science*, having had the patience to read the first three volumes of Dr. Zollner (the fourth not having arrived in Paris when I wrote my little volume).

That which most surprises us concerning these matters, as Dr. Lankaster wittily observed before the criminal tribunal, is not the habitual practices of the trickster Slade, but that they found gentlemen of intelligence who testified to the *bona fides* of these exhibitions.

O poor human reason! what absurdities are uttered in thy name! And yet notwithstanding its weaknesses, of which we see so many proofs all about us, what marvels hast thou not discovered, what progress made, thanks to those admirable faculties with which the Creator in his providence endowed man, when in His own image he created him.

The cause, the sole cause, of these weaknesses is explained by the course of the wiseacres themselves, who do not demand sufficient proof as did Newton that the earth is attracted by the sun ; they should go forth and find the secret of nature and determine the cause of the attraction.

It is in those puerile researches conducted, with so much vanity, that their judgment fails, and ignoring the dimensions of Euclid, they are induced to adopt those of Dr. Zollner, which reason rejects.

They indulge in this gratuitous hypothesis, a kind of entrancement which assigns to space a fifth, and even, as we have seen, a fractional number of dimensions. Thus we are opposed by a battery of empty phrases, made up of incomprehensible words, and recourse is had to the knots and slates of Dr. Slade in support of their theory. By force of such reasoning they are led to dabble in miracles, whilst those who invoke them do not use their reason at all.

They even forget the admirable precept of Horace, who in his journey to Brindes encountered priests, spirits and charletons who showed him a resinous material which they liquified at pleasure, through the intermediation of their idol. If they had a knot to untie, to resolve or to cut, they appealed to the spirit of Mécene, imploring that the knot should be deemed worthy of his intervention. If you employ a god to accomplish the miracle, it is clear that the miracle should be wrought neither at the foundation of a provincial sanctuary nor in the midst of darkness, but the heavens should be the magnificent theatre where your god displays his power and his majesty.

Is it not humiliating to see in the place of these ancient teachings, which we are too ready to renounce ; the theory of the slate and the knots of Dr. Slade ? If the voyage to Brindes were to be more generally read, we should hear less often of certain impostors whose repute shows to what lengths of retrogation cultivated minds sometimes permit themselves to be carried.

Fortunately the lives of certain great men illustrate to what heights the genius of our race is capable of elevating itself, and the spirit of

Mécene may at some future period resemble Homer, of whom, after three thousand years, it is said he is still young in glory and immortality.

---

The foregoing is a rather extravagant presentation of the fraud theory, in respect to those peculiar manifestations which have so long puzzled the curious and defied the scrutiny of such of the learned as entered the field of enquiry with a predetermined effort at exposure. But the more candid of this class of investigators will surely admit that, taken as a whole, the manifestations which occur in the presence of Mr. Slade are quite distinct from those of the prestidigitator, who is abundantly supplied with ingenious trick boxes, tables, and miscellaneous paraphernalia to help out his wonder-working illusions, whilst, notwithstanding Dr. Lankaster's contrary statements, Mr. Slade resorts to no such aids to deception.

A native of New York State, Mr. Slade, in early childhood, developed *mediumship* by which term is commonly understood a means through his organism of spirit communion with mortals. The query is, are the various manifestations which occur in Mr. Slade's presence, the work of invisible agencies, acting in a manner independently of him, or are they the result of trick ?

A leading feature of Mr. Slade's mediumship or performance, as the case may be, is what is termed chirography, or independent writing, usually upon slates, but not necessarily so. There are various other phases of manifestation which cannot be explained upon the theory of imposture. Such an attempt would be even more ridiculous than the explanation which is given by M. de Fonvielle of the *modus operandi* of slate writing, to which he has prudently confined himself. It is clear that he has not approached this subject in anything like a fair and candid spirit. He first endeavors to cast discredit upon Mr. Slade by raking up his differences with Dr. Lankaster, and his temporary conviction by an English police magistrate as far back as 1876.

He next endeavors to belittle Prof. Zollner, and questions the honesty of his motive in entering upon these occult experiments. The truth of the matter is that Mr. Slade has served as a public medium almost from childhood, and is well and favorably known in all the leading States of this Union, as a most extraordinary medium, through whose instrumentality marvelous psychic phenomena have been presented, including independent slate writing in several languages, at one and the same time, and the moving of ponderous objects without visible contact.

His engagement with the Leipsic professors was purely for scientific purposes. The party included Professors Zollner, Weber, Scheibner and Fichner, all eminent scientists and gentlemen of the highest repute. There can be no excuse for this concealment of important facts, since M. de Fonvielle has taken pains to advise us of his familiarity with Prof. Zollner's published account of those sittings, in which all these names appear in solemn affirmation of its reliability.

Thus it will be seen that M. de Fonvielle has "*falsified the record*," and having in this way established his premises, he proceeds to demolish his man of straw, and advertise his little book. This is all there is of it: he does not reason at all, rightly, and there is nothing for us to answer or contradict in defence of our countryman or the Leipsic Professors, but his misrepresentations. He even goes so far as to apply an offensive epithet to Professor Zollner, hardly translatable, and accuse him, now that he has passed to another life, of being actuated by an envious rivalry of contemporary English scientists. That our readers may not be mislead, we will state that Professor Zollner occupied, at that period, a front rank among the ablest savants of Europe, and filled the chair of physics and astronomy at the Leipsic University. He was also a member of the leading scientific societies of Europe and Great Britain, and was confessedly the peer of the foremost of his colleagues. Moreover, he was a *materialist*, and could have had no disagreement with any of them which his changed attitude towards occult phenomena, would not have healed rather than intensified.

Prof. Weber, then upwards of seventy years of age, was Professor of Physics; the founder (with his brother) of the doctrine of the Vibration of Forces, and author of a standard work, in four volumes, on Electro-Dynamic Measurement.

Prof. Fechner, born in 1801, was also Professor of Physics at Leipsic, and author of several scientific works, including "The Soul of Plants," "The Zendavesta," "Elements of Psycho-Physics and the Problems of the Soul."

Prof. Scheibner was a distinguished Professor of Mathematics at the Leipsic University.

Such were the aids of Prof. Zollner in his investigations. How do these facts compare with M. de Fonvielle's statement? In the course of his article he alludes only to slate writing and knot tying, when, in fact, phenomena of much greater magnitude occurred in the presence of these distinguished men, whose investigations were continued for several weeks and ran well on to a hundred sittings. In his published report of them, Prof. Zollner has included illustrations and fac-similes of the plan and results of some of the more remarkable experiments, in which occurred the passage of matter through matter, and the mysterious disappearance of a small table and its subsequent return as from the ceiling of the room. The plan of gaining a bad case by misrepresenting the testimony and abusing the opposite counsel, is quite out of fashion in intelligent communities. Were it an object to prove the existence of steam motors, electric telegraphs and telephones, and the theory of storms practically applied as a means of saving life and property, we should not go to "Horace" for enlightenment, but to these masterly achievements of genius themselves which adorn the age in which we live. The investigation of a single day among these living facts, would be more profitable to this end than a whole lifetime spent among the tombs of antiquity. To intrench one's self in his own self-satisfied ignorance, and not only refuse to be enlightened, but to deny the evidences by means whereof more advanced minds have gained the knowledge of a great truth, is among the worst exhibitions of bigotry. M. de Fonvielle has presented no facts within his own knowledge or the affirmation of others, which point to Mr. Slade as a trickster, save the single one of his arrest more than ten years ago, at the instigation of Dr. Lankaster, with whom Mr. Slade, immediately after his release, offered to undergo any reasonable test of his honesty, but this offer met with no response. Was Dr. Lankaster fearful that he would be "convinced against his will?"

We have thus far confined our remarks to Mr. Slade, and the phenomena of which he is the instrument, because M. de Fonvielle covers these points only, but if any proof of the genuineness of these and similar manifestations were wanting, witnesses could be brought forward by thousands to furnish it out of their own experiences. Mr. William Eglinton, of London, Eng., has but recently overwhelmed one of his defamers with a superabundance of testimonials from gentlemen and ladies of the highest standing, including lawyers, doctors, scientists and clergymen, in affirmance of the verity of phenomena which are common in his presence, including chirography. In the United States, mediums of these phenomena are to be met with everywhere. They are to be found, not only in the seance room accessible to the public, but largely in private families, whose members, in this way, have the truth brought home to them. Thus it is that the work of proselyting is quietly going on, and men and women, of all classes, are fast being brought to a *knowledge* of the after-life which no argument is able to undermine.

Does M. de Fonvielle profess an intimate acquaintance with the prestidigitator's art, or a keener eye and more dexterous hand than Houdin, Jacobs, Bellachini, Hermann or Keller, the acknowledged masters of it? These have severally confessed, after the most searching scrutiny, that the manifestations which occur in presence of mediums are beyond their ken; that so far as they were able to perceive, there was no element of fraud or trickery about them?

Is not this enough, or shall we grope our way with M. de Fonvielle into the darkness of remote periods; when superstition wrapped as with a pall the varied centuries, to bring from the tomb of the buried past, a ghostly interpreter of the present, that speaks in a language that is dead, and knows not of the sun-lit heights attained by the living? Well may we exclaim with M. de Fonvielle, "O, poor human reason, what absurdities are uttered in thy name!"

